# Neurovascular and Immuno-Imaging: From Mechanisms to Therapies. Proceedings of the Inaugural Symposium

**DOI:** 10.3389/fnins.2016.00046

**Published:** 2016-02-22

**Authors:** Katerina Akassoglou, Dritan Agalliu, Christopher J. Chang, Dimitrios Davalos, Jaime Grutzendler, Elizabeth M. C. Hillman, Baljit S. Khakh, David Kleinfeld, Dorian B. McGavern, Sarah J. Nelson, Berislav V. Zlokovic

**Affiliations:** ^1^Gladstone Institute of Neurological Disease, University of California, San FranciscoSan Francisco, CA, USA; ^2^Department of Neurology, University of California, San FranciscoSan Francisco, CA, USA; ^3^Departments of Neurology, Pathology and Cell Biology and Pharmacology, Columbia University Medical CenterNew York, NY, USA; ^4^Departments of Chemistry and Molecular and Cell Biology, Howard Hughes Medical Institute, Helen Wills Neuroscience Institute, University of California, BerkeleyBerkeley, CA, USA; ^5^Department of Neurosciences, Lerner Research Institute, Cleveland Clinic FoundationCleveland, OH, USA; ^6^Department of Neurology, Yale UniversityNew Haven, CT, USA; ^7^Laboratory for Functional Optical Imaging, Departments of Biomedical Engineering and Radiology, Kavli Institute for Brain Science, Columbia UniversityNew York, NY, USA; ^8^Departments of Neurobiology and Physiology, David Geffen School of Medicine, University of California, Los AngelesLos Angeles, CA, USA; ^9^Department of Physics and Section of Neurobiology, University of California, San DiegoLa Jolla, CA, USA; ^10^Viral Immunology and Intravital Imaging Section, National Institute of Neurological Disorders and Stroke, National Institutes of HealthBethesda, MD, USA; ^11^Department of Radiology and Biomedical Imaging, University of California, San FranciscoSan Francisco, CA, USA; ^12^Department of Physiology and Biophysics, Keck School of Medicine, Zilkha Neurogenetic Institute, University of Southern CaliforniaLos Angeles, CA, USA

**Keywords:** neuroinflammation, multiple sclerosis, traumatic brain injury, Alzheimer's disease, blood-brain barrier, microglia, myelin, two-photon microscopy

## Abstract

Breakthrough advances in intravital imaging have launched a new era for the study of dynamic interactions at the neurovascular interface in health and disease. The first Neurovascular and Immuno-Imaging Symposium was held at the Gladstone Institutes, University of California, San Francisco in March, 2015. This highly interactive symposium brought together a group of leading researchers who discussed how recent studies have unraveled fundamental biological mechanisms in diverse scientific fields such as neuroscience, immunology, and vascular biology, both under physiological and pathological conditions. These Proceedings highlight how advances in imaging technologies and their applications revolutionized our understanding of the communication between brain, immune, and vascular systems and identified novel targets for therapeutic intervention in neurological diseases.

## Introduction

**Figure 1:**
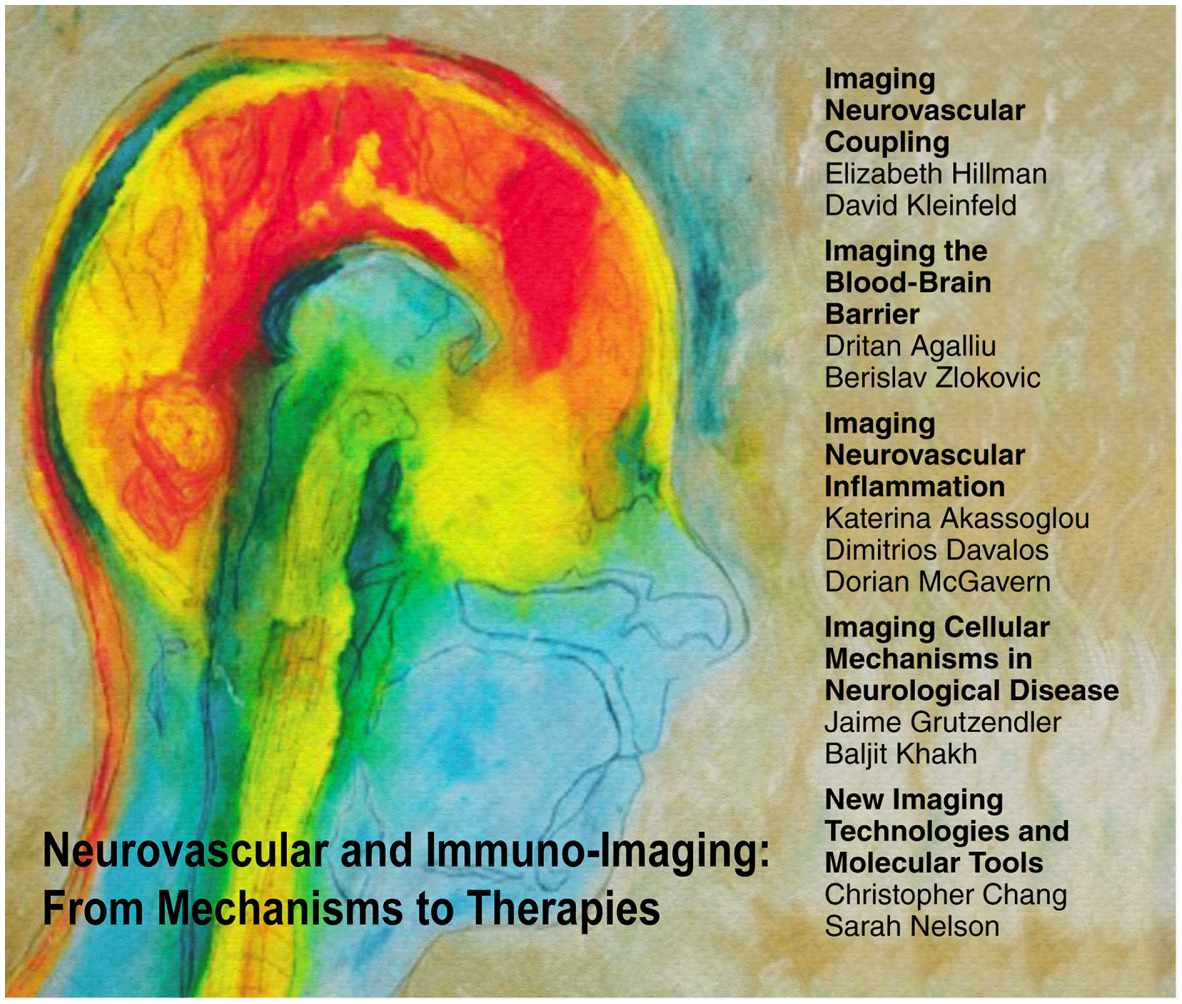
**Five thematic areas discussed at the Neurovascular and Immuno-Imaging Symposium**. Etching entitled “Mind on Fire” kindly provided by Elizabeth Jameson.

The presentations at the first Neurovascular and Immuno-Imaging Symposium covered a broad range of topics, from physiological neurovascular coupling in the brain to cellular and molecular responses at the blood brain barrier (BBB), to recent developments in molecular imaging, bioengineering, and the identification of novel therapeutic targets for human disease. A common thread among the presentations was the study of the nervous system in health and disease using imaging technologies and molecular tools for recording ongoing biological processes in living organisms, from mice to humans (Figure 1). Brief outlines of the presentations together with a discussion of the imaging approaches used and the implications of the findings for better understanding of normal brain function or pathological processes in disease, have been grouped in the following five thematic areas.

## Imaging neurovascular coupling

Physiological brain function relies on a large number of interconnected cellular, metabolic, and vascular processes. The term *neurovascular coupling* describes the relationship between neural activity and metabolism with alterations in cerebral blood flow (CBF). Though the interaction among these processes is well established, the mechanisms linking them and their importance for brain function remain largely unknown. Functional neurovascular coupling refers to the local modulation of CBF that occurs at sites of neural activity in the brain (Kozberg et al., 2013; Hillman, 2014).

Many brain cell types link neural activity and vessel dilation, including astrocytes (Takano et al., 2005), interneurons (Cauli et al., 2004), and pericytes (Hall et al., 2014). However, many features of the cortical hemodynamic response to neural activity are unaccounted for by proposed models. Dr. Elizabeth Hillman presented work from her group, demonstrating that the vascular endothelium plays a role in this active coupling process by propagating vasodilatory signals upstream to pial arterioles (Chen et al., 2014). This pathway, which is known to provide local blood flow modulation elsewhere in the body, has many component mechanisms with different spatiotemporal properties and pharmacological sensitivities (e.g., COX and NO dependent and independent mechanisms; Wölfle et al., 2011) and can explain many of the previously anomalous results of earlier studies. Moreover, dependence of neurovascular coupling on healthy endothelial function implicates a far wider range of systemic cardiovascular disorders as having direct cerebrovascular impact. A state in which endothelial coupling is impaired could lead to neurodegeneration, or even acute cognitive deficiencies. Equally, drugs, such as those aimed at treating blood pressure, inflammation and pain, which act directly on pathways within the vascular endothelium, might affect neurovascular coupling (Bakalova et al., 2002).

Dr. David Kleinfeld presented optical imaging studies that could explain reported resting-state functional magnetic resonance imaging (fMRI) observations correlating neuronal activity in different parts of the brain and slow alterations in blood oxygen levels. *fMRI* is a powerful tool to probe the extent of activity in the human brain, and the blood-oxygenation-level-dependent (BOLD) fMRI signal (Ogawa et al., 1990) in particular, forms the central technology of modern cognitive neuroscience. An intriguing issue is that ultra-slow variations (~0.1 Hz) in brain tissue oxygenation appear mirrored across conjugate brain areas of the two hemispheres (Biswal et al., 1995). The discovery of this “resting-state” BOLD fMRI (Fox and Raichle, 2007) has been inverted in human cognition studies, so that ultra-slow co-fluctuations are interpreted as *function connections* (Sporns et al., 2005). Yet a mechanism explaining this observation has been lacking. The Kleinfeld group used ultra-large field two-photon laser scanning microscopy (Tsai et al., 2015) in mice with a thin-skull transcranial window (Drew et al., 2010), together with conventional techniques, to perform microscopic measurements of neuronal activity, vascular dynamics, and tissue oxygenation. They discovered evidence for a biophysical basis linking the co-activation of ongoing neuronal activity with ultra-slow oscillations in blood oxygenation that could justify inferring neuronal connections from synchronous ultra-slow vasodynamics across different brain areas.

## Imaging the blood-brain barrier

Neuronal computation and normal brain function requires tight control of the chemical composition of the neuronal “milieu” that is maintained by the BBB (Zlokovic, 1995; Zlokovic et al., 2010). Brain endothelial cells tightly interconnected through adherens and tight junctions (TJs) are the building blocks of the barrier, while astrocytes and pericytes are essential for BBB formation and maintenance. The BBB plays a pivotal role for the healthy central nervous system (CNS) as it regulates the access of blood-borne solutes to the brain and spinal cord, and limits the entry of neurotoxic plasma-derived proteins, circulating metals, red blood cells and leukocytes into the brain.

Dr. Berislav Zlokovic discussed the role of pericytes for BBB breakdown, particularly in the context of neurodegenerative disease. Studies from his group in transgenic murine models have shown that chronic BBB breakdown caused by pericyte degeneration or aberrant signaling to pericytes from endothelial cells or astrocytes leads to accumulation of blood-derived neurotoxic proteins in the CNS (e.g., fibrin, thrombin, hemoglobin, free iron, plasmin; Bell et al., 2010, 2012). This causes progressive neurodegeneration mediated by direct neuronal toxicity, oxidant stress and/or detachment of neurons from the extracellular matrix (Bell et al., 2010, 2012). Pericytes degenerate in Alzheimer's' disease (AD) that is associated with BBB breakdown in human post-mortem studies (Sengillo et al., 2013; Halliday et al., 2016). Accelerated pericyte degeneration in a murine model of AD leads to BBB breakdown and increased accumulation of Alzheimer's toxin amyloid-β (Sagare et al., 2013). Using a high resolution dynamic contrast-enhanced magnetic resonance (MR) imaging protocol to quantify regional BBB permeability in the human brain, they showed BBB breakdown during normal aging that begins in the hippocampus (a region critical for learning and memory), worsens with mild cognitive impairment, and correlates with pericyte injury (Montagne et al., 2015).

Although BBB breakdown is a hallmark of many neurological disorders like AD, ischemic stroke and multiple sclerosis (MS; Zlokovic, 2008), the cellular mechanism of BBB disruption in different diseases is not established. In the healthy CNS, endothelial cells regulate both paracellular and transcellular access through the BBB via the presence of TJs and scarce endocytotic vesicles, respectively. Dr. Dritan Agalliu presented his group's studies of structural and functional BBB changes using *in vivo* two-photon microscopy in animal models of ischemic stroke and neuroinflammatory disease. Using a novel transgenic mouse strain with eGFP-labeled TJs (*Tg eGFP-Claudin5*) they found that TJs break down between 24 and 48 h after occlusion in the transient Middle Cerebral Artery Occlusion animal model (t-MCAO). However, BBB function was impaired as early as 6 h after stroke, a time point at which they found an increased rate of transcytosis within endothelial cells (Knowland et al., 2014). These findings suggest a stepwise impairment in transcellular followed by paracellular pathways that contribute to BBB breakdown in ischemic stroke. Moreover, Caveolin-1 deficient mice, that have reduced transcellular permeability, display a normal increase in paracellular permeability after t-MCAO, suggesting that these two mechanisms are independent (Knowland et al., 2014). In similar studies in the MS animal model Experimental Autoimmune Encephalomyelitis (EAE), they found that recycling of TJ proteins was faster in the spinal cord than in the cortex, which may underlie the preferential vulnerability of the spinal cord to EAE. Moreover, dynamic changes in TJs occur prior to EAE onset and throughout the disease. In contrast, BBB transcytosis increases at peak EAE and *Caveolin1*^−∕−^ mice exhibit decreased peak severity and demyelination. These findings suggest that TJ disruption is necessary for the onset of EAE, whereas transcellular permeability enhances disease severity. Moreover, the kinetics of paracellular vs. transcellular impairment in BBB function is quite different between ischemic stroke and EAE.

## Maging neurovascular inflammation

BBB disruption, microglial activation and neurodegeneration are hallmarks of MS (Lassmann et al., 2001; Dutta and Trapp, 2014). However, whether plasma proteins contribute to neuroinflammation and neuronal damage remains poorly understood. Studies in human MS have identified abundant deposition of the blood coagulation factor fibrinogen, not only in active and chronic plaques, but also in early lesions prior to demyelination (Kwon and Prineas, 1994; Claudio et al., 1995; Gay et al., 1997; Vos et al., 2005; Marik et al., 2007; Han et al., 2008). Dr. Katerina Akassoglou discussed her group's studies that identified fibrinogen-induced microglial activation as a critical mediator of axonal damage in neuroinflammation (Adams et al., 2007; Davalos et al., 2012, 2014; Bardehle et al., 2015). Using *in vivo* two-photon microscopy, they showed that in EAE, microglia specifically cluster around blood vessels with BBB disruption at sites of perivascular fibrin deposition even prior to disease onset (Davalos et al., 2012). By developing a molecular probe to detect coagulation activity in the CNS, they demonstrated early activation of coagulation and fibrin deposition even before onset of EAE (Davalos et al., 2014). Intriguingly, fibrin is sufficient to induce reactive oxygen species release by microglia, and thus contribute to neurodegeneration (Davalos et al., 2012). Pharmacologic or genetic disruption of the interaction between fibrin and the CD11b/CD18 integrin receptor (other names Mac-1, complement receptor 3) on microglia suppresses microglial cluster formation, neurological symptoms, inflammation, demyelination, and axonal damage in EAE (Adams et al., 2007; Davalos et al., 2012). Overall, their studies identified fibrinogen as a novel activator of CNS innate immunity that promotes neurodegeneration. Fibrin has the potential for selective drug targeting to suppress its damaging functions in the nervous system without affecting its beneficial effects in hemostasis (Adams et al., 2007; Davalos and Akassoglou, 2012; Davalos et al., 2012; Bardehle et al., 2015; Ryu et al., 2015). Fibrin-selective inhibition of innate immunity may offer novel strategies to combat axonal damage in neuroinflammatory disease.

BBB disruption through the compromise of the TJs of the non-fenestrated endothelium comprising the cerebral and meningeal blood vessels can also be the result of CNS infections and injuries. The resulting unregulated passage of blood-derived materials into the cerebrospinal fluid and CNS parenchyma can in turn give rise to neurological dysfunction, seizures, edema, and ultimately, fatal herniation if unrelieved. Dr. Dorian McGavern discussed recent studies showing that both mechanical and immunological factors can contribute to alterations in the integrity of CNS vasculature (Kim et al., 2009; Roth et al., 2014). Following traumatic brain injury (TBI), mechanical forces stemming from the injury itself can open and / or occlude CNS vasculature, even when the injury is mild (Roth et al., 2014; Corps et al., 2015). This can trigger regional hypoxia, edema, and cell death, resulting in the mobilization of a sterile immune reaction driven by purinergic receptor signaling. Moreover, CNS infection by viruses, bacteria, parasites, and fungi can similarly open cerebral vasculature, yet through different mechanisms (Kang and McGavern, 2010). Meningitis and encephalitis are commonly observed following CNS infection and result from the recruitment of immune cells into the meninges or parenchyma, respectively (Swanson and McGavern, 2015). Innate and adaptive immune cells are recruited to the CNS to clear invading pathogens, but sometimes the manner by which these cells are recruited promotes vascular injury. Intravital imaging studies by his group revealed that pathogen-specific CD8+ T cells can promote synchronous extravasation of innate myelomonocytic cells through release of chemoattractants (Kim et al., 2009; McGavern and Kang, 2011). This type of extravasation is incredibly injurious to CNS vasculature and contributes to seizures and fatal edema. Thus, while the mechanisms of vascular breakdown may differ between infections and injuries, the resultant neurological complications stemming from a loss in CNS barrier integrity are often similar.

## Imaging cellular mechanisms in neurological disease

Dr. Jaime Grutzendler discussed novel methods for longitudinal imaging of the brain microvasculature and myelinated axons *in vivo* and presented new biological discoveries made with such techniques. The fibrinolytic system has been recognized as the principal mechanism in charge of clearing the vasculature from occluding thromboemboli. Two photon microscopy of cortical capillaries *in vivo* revealed an additional innate mechanism of microvascular recanalization capable of clearing emboli composed of a variety of substances including those not susceptible to fibrinolysis (Lam et al., 2010). This mechanism which they termed *angiophagy* involves rapid remodeling of the endothelium, engulfment of the occluding emboli and their translocation through the arteriolar vascular wall (Lam et al., 2010; Grutzendler et al., 2014). This discovery has the potential to greatly impact our understanding and treatment of thromboembolic disorders and the ischemia no-reflow phenomenon. His group also developed a label-free technique (Spectral Confocal Reflection Microscopy-SCoRe) to image myelinated axons *in vivo* (Hill and Grutzendler, 2014; Schain et al., 2014). This technique allowed for the first time high-resolution *in vivo* imaging of brain myelin development and pathology in mice and is being explored for potential *in vivo* diagnostic applications in peripheral nerve human disorders. This method opens new capabilities to study myelin development and pathology and functional interactions with neurovascular cells in living mice and other animals.

Huntington's disease (HD) is caused by intracellular accumulation and aggregation of a mutant form of the huntingtin protein (mHTT). Dr. Baljit Khakh discussed his group's recent findings that suggest neural circuit–specific astrocyte roles for the onset and progression of disease in HD mouse models. They found that a significant increase in astrocyte mHTT is associated with a reduction of important functional proteins like Glt1 (EAAT2) and Kir4.1, which alter astrocyte function without noticeable astrogliosis at least at the onset of neurological symptoms (Tong et al., 2014). Kir4.1 potassium channels are expressed primarily by astrocytes and regulate extracellular K^+^ levels (Kofuji and Newman, 2004) in the CNS, and loss of the glutamate transporter Glt1 (EAAT2) has been extensively studied in the context of HD (Estrada-Sánchez and Rebec, 2012). Indeed, loss of Kir4.1 in striatal astrocytes led to higher ambient extracellular K^+^ concentrations, which contributed to increased medium spiny neuron (MSN) excitability in HD mouse models (Tong et al., 2014). Moreover, selective expression of GFP-labeled Kir4.1 channels in astrocytes rescued these deficits, demonstrating that astrocyte Kir4.1 loss can phenocopy hallmark MSN changes of HD mouse models (Cepeda et al., 2010). Together these results support that key aspects of altered MSN excitability in HD are caused at least in part by the failure of astrocytes to maintain homeostatic levels of extracellular K^+^. This would imply an important yet diverse role for astrocytes in HD pathology, which changes with the disease progression. At early stages of the disease deficits in Kir4.1 and Glt1 (EAAT2) could contribute to underlying HD pathological mechanisms, which could further facilitate the progressive increase in astrocyte reactivity observed with disease progression in humans. Thus, therapeutic strategies targeting astrocytes need to take into account a range of potential astrocytic contributions to HD progression ranging from early homeostatic dysfunction to advanced reactive responses.

## New imaging technologies and molecular tools

MR is a powerful non-invasive imaging modality that makes it possible to visualize anatomic and vascular structures in the brain, as well as physiological and metabolic properties that reflect changes in biological function. Dr. Sarah Nelson discussed recent advances in technology that allow whole body 7T MR scanners and high performance gradients, providing major improvements in the signal to noise ratio and sensitivity achieved in the human brain. High resolution time of flight angiography and susceptibility weighted imaging have been used to take advantage of these capabilities and are critical for monitoring changes in arterial and venous structures associated with vascular disease, aging, trauma and radiation-induced damage (Lupo et al., 2012). The contrast obtained with blood oxygenation level dependent (BOLD) and phase imaging is significantly higher at 7T, facilitating improved fMRI and the detection of abnormalities in deep gray matter structures that are associated with neurodegenerative diseases such as AD, HD (Apple et al., 2014) and MS (Bian et al., 2013). Metabolic imaging that uses multi-voxel H-1 spectroscopy has been applied to evaluate abnormalities caused by neurological and psychiatric diseases and has been shown to benefit from both the higher signal to noise ratio and increased spectral resolution that is possible at 7T (Li et al., 2015) MR imaging.

Dr. Christopher Chang focused his presentation on the chemistry/neuroscience interface, and in particular on the development of molecular imaging probes to enable discovery and study of new types of chemical signals based on metals and redox molecules that govern brain activity. The traditional view of metals in biology is that sodium, potassium, and calcium are the major dynamic signals whereas transition metals are solely static metabolic cofactors. However, invention of several classes of fluorescent probes for metals (Domaille et al., 2008; Que et al., 2008; Aron et al., 2015; Cotruvo et al., 2015) led to the identification of copper as an endogenous regulator of spontaneous activity (Dodani et al., 2011, 2014), representing a new paradigm of transition metal signaling (Chang, 2015). Along the same lines, Dr. Chang presented work from his group illustrating an alternative approach to sensing where reactive small-molecule analytes are sorted by their chemical reactivity as opposed to shape-based recognition and binding (Chan et al., 2012). New probes for emerging signaling / stress molecules like hydrogen peroxide (Lippert et al., 2011) and hydrogen sulfide (Lin et al., 2015) have been devised to identify the essential role of redox molecules in neural stem cells (Dickinson et al., 2011) and H_2_O_2_/H_2_S crosstalk (Lin et al., 2013). These examples illustrate the utility of chemoselective probes for selective molecular imaging of biologically relevant molecules in their native environments.

## Concluding remarks

The first Neurovascular and Immuno-Imaging Symposium provided a forum for the discussion of current research on the cellular and molecular mechanisms that regulate the interface between brain and periphery, both in physiology and in neurological disease. Imaging technologies have proven invaluable in facilitating new discoveries that have reshaped our understanding of cellular behaviors in the CNS (Davalos and Akassoglou, 2008; Merlini et al., 2012). Such discoveries have began to explain not only fundamental physiological functions like the coupling of neuronal activity to blood supply in the brain, but also underlying mechanisms for disease development, like those when the BBB is compromised and peripheral immune cells infiltrate the CNS. The continuous development of new imaging technologies and novel molecular tools that can target ongoing cellular and molecular events at the neurovascular interface will further expand our capabilities to decipher physiological and pathological mechanisms, and design better strategies for therapies in neurological diseases.

## Author contributions

All authors listed, have made substantial, direct and intellectual contribution to the work, and approved it for publication. KA organized and chaired the Symposium. DD and KA wrote the manuscript with contribution from all authors.

### Conflict of interest statement

The authors declare that the research was conducted in the absence of any commercial or financial relationships that could be construed as a potential conflict of interest.
